# Visualising mouse neuroanatomy and function by metal distribution using laser ablation-inductively coupled plasma-mass spectrometry imaging†
†Electronic supplementary information (ESI) available: Supplementary figures, tables and videos. See DOI: 10.1039/c5sc02231b



**DOI:** 10.1039/c5sc02231b

**Published:** 2015-07-27

**Authors:** Bence Paul, Dominic J. Hare, David P. Bishop, Chad Paton, Van Tran Nguyen, Nerida Cole, Megan M. Niedwiecki, Erica Andreozzi, Angela Vais, Jessica L. Billings, Lisa Bray, Ashley I. Bush, Gawain McColl, Blaine R. Roberts, Paul A. Adlard, David I. Finkelstein, John Hellstrom, Janet M. Hergt, Jon D. Woodhead, Philip A. Doble

**Affiliations:** a School of Earth Sciences , The University of Melbourne , Parkville , Victoria 3052 , Australia; b The Florey Institute of Neuroscience and Mental Health , The University of Melbourne , Parkville , 3052 , Victoria , Australia; c Elemental Bio-imaging Facility , University of Technology Sydney , Broadway , 2007 , New South Wales , Australia . Email: dominic.hare@uts.edu.au ; Email: philip.doble@uts.edu.au ; Tel: +61 2 9512 1792; d Senator Frank R. Lautenberg Environmental Health Sciences Laboratory , Department of Preventive Medicine , Icahn School of Medicine at Mount Sinai , New York , 10029 , New York , USA; e Centre for Star and Planet Formation , Geological Museum , University of Copenhagen , Øster Voldgade 5-7 , DK-1350 Copenhagen , Denmark

## Abstract

Metals have a number of important roles within the brain. We used laser ablation-inductively coupled plasma-mass spectrometry (LA-ICP-MS) to map the three-dimensional concentrations and distributions of transition metals, in particular iron (Fe), copper (Cu) and zinc (Zn) within the murine brain. LA-ICP-MS is one of the leading analytical tools for measuring metals in tissue samples. Here, we present a complete data reduction protocol for measuring metals in biological samples, including the application of a pyramidal voxel registration technique to reproducibly align tissue sections. We used gold (Au) nanoparticle and ytterbium (Yb)-tagged tyrosine hydroxylase antibodies to assess the co-localisation of Fe and dopamine throughout the entire mouse brain. We also examined the natural clustering of metal concentrations within the murine brain to elucidate areas of similar composition. This clustering technique uses a mathematical approach to identify multiple ‘elemental clusters’, avoiding user bias and showing that metal composition follows a hierarchical organisation of neuroanatomical structures. This work provides new insight into the distinct compartmentalisation of metals in the brain, and presents new avenues of exploration with regard to region-specific, metal-associated neurodegeneration observed in several chronic neurodegenerative diseases.

## Abstract 2

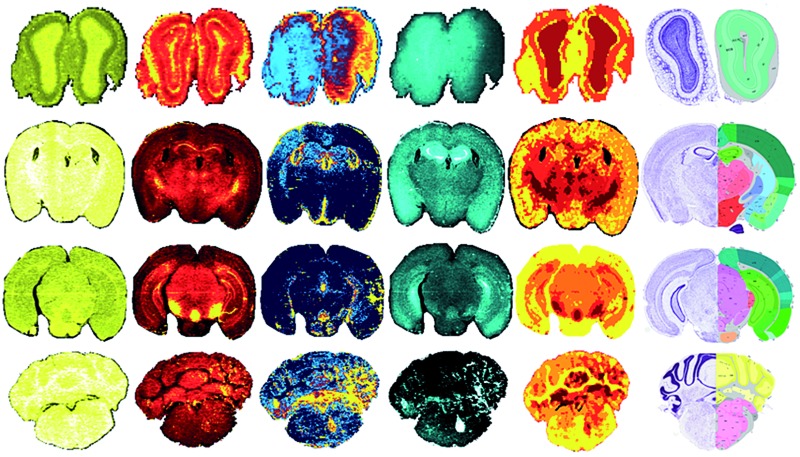
Studying the neuroanatomy of the mouse brain using imaging mass spectrometry and chemometric analysis.

## Introduction

It is estimated that nearly half of all enzymes require a metal ion to function.^1^ Redox-active transition metals are at high concentrations in the brain, where they mediate metabolic processes that account for approximately 20% of the body's total energy consumption.^2^ Transition metals, including manganese (Mn), iron (Fe), cobalt (Co), copper (Cu) and zinc (Zn), have important roles as cofactors in enzymes responsible for normal neural functioning.^3^ In a number of neurodegenerative diseases, particularly those associated with ageing, metal homeostasis is disrupted, resulting in unchecked redox activity and increased oxidative stress.^4^

Neuroanatomical atlases have evolved significantly since the seminal *Rat Brain in Stereotaxic Coordinates* by Paxinos and Watson was published in 1982.^5^ Nearly every neuroscience laboratory using rodent models still uses this resource, though in recent years there has been a trend toward atlases that align anatomical features with their biological functionalities, such as those based on regional gene expression patterns and the neural ‘connectome’.^6^ Considering the importance metals play in a wide range of neurological processes, visualising the spatial distribution of metals in the brain is crucial for both understanding their role in basic neural function and elucidating the mechanisms by which they can impart toxicity. As yet, a ‘metallomic’ brain atlas has not been developed.

As the first step toward a standard model of metal distribution in the murine brain, here we present a new approach for the mapping of metal distributions in this well-studied animal model. Several techniques are available for this task,^7^ including histochemical staining (which is typically only qualitative),^7^ synchrotron-based X-ray fluorescence microscopy (XFM; which is limited only by accessibility to the approximately 40 synchrotrons worldwide)^8^ and laser ablation-inductively coupled plasma-mass spectrometry (LA-ICP-MS),^9,10^ which is the focus of this study.

The ubiquity of metals throughout the brain, as well as the inter-animal variability in total metal content, present several major hurdles to this task. Firstly, how can we directly associate metal species with metal-binding proteins, which ultimately dictate the biological function of a metal? Direct labelling of antibodies with metal tags provides an innovative means to image antigen distributions; for example, β-amyloid distribution in a transgenic mouse model of Alzheimer's disease was visualised by LA-ICP-MS using europium-tagged antibodies.^11^ When endogenous metals and biomolecules are simultaneously imaged and quantified, we can explore the associations between metal location and protein function.^12^ Secondly, metal distributions in the brain are highly influenced by metal exposures from the environment (such as through drinking water), even in a relatively controlled system such as an animal house. Robust analytical methods are necessary to profile inter-animal variability, so that the ‘typical’ metal concentration within specific brain nuclei can be reliably referenced and used as a resource for identifying aberrant metal homeostasis in mouse models of disease.

**Fig. 1:**
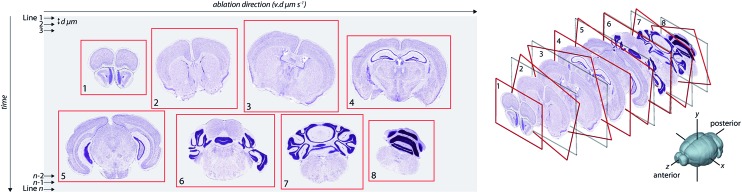
Image transformation and stacking. Imaging by LA-ICP-MS rasters a laser beam across the sample surface, creating a two-dimensional image by reduction of individual lines of ablation into a single data file. Total acquisition time increases with decreasing spot size. Cut tissue sections, mounted with irregular orientation, are background corrected and subdivided into individual images, anterior to posterior. These images are then aligned using a pyramidal pixel registration technique to produce a voxelgram with spatial dimensions dictated by laser spot size (*y*-direction), laser scan speed (*x*-direction) and spacing between sections (*z*-direction).

Visualisation of metal distributions in brain tissue is usually achieved using two-dimensional imaging, which may lose structural features that could be of importance. Three-dimensional imaging by LA-ICP-MS has been used to assess metal distribution in irregularly-shaped brain regions that may otherwise be misrepresented by traditional 2D mapping.^9,10,13^ However, alignment of consecutive images prepared using typical histology protocols relies upon manual registration^14^ or simple affine transformation,^15^ which do not account well for morphological changes as one moves through an organ with complex architectures. Unlike emission-based imaging (*e.g.* XFM tomography), where an intact sample is rotated within the confines of the microprobe and a comparatively simple linear transform is used to determine the central rotational axis for slice alignment,^16^ reconstruction of LA-ICP-MS images in three-dimensional space requires reassembly of individually-cut sections. Reassembly of the metal content of each section into a quantitative three-dimensional image requires an alignment strategy that can register a dynamic and complex structure whilst ensuring that instrument variables (such as signal drift or instability) are taken into account, which is a considerable analytical challenge (Fig. 1).

Here, we describe a novel data reduction protocol for the reconstruction and interpretation of three-dimensional LA-ICP-MS images, which is based on a pyramidal voxel approach to reproducibly align tissue sections. For the implementation of our protocol, we developed an interactive user interface derived from the ‘Iolite’ software package used primarily in the geosciences,^17^ which we have called ‘Biolite’. Our approach to three-dimensional reconstruction is more mathematically robust than alternative methods for LA-ICP-MS imaging, and is less prone to user bias.^13^ We present the reconstruction of redox-active metal distributions within the complete mouse brain, and how discrete regions of metal distribution correspond to specific areas of neurological functionality. Finally, to demonstrate the potential of this technology, we have applied this imaging approach to study the brain-wide co-localisation of Fe and dopamine, a potent redox couple implicated in parkinsonian neurodegeneration.^12^

## Results

### Relative quantification between samples

Serial sections of cryopreserved male C57BL/6 mouse brains were scanned in batches; 30 μm-thick sections were mounted on a standard microscope slide and an equivalent ablation area (approx. 22 × 60 mm) was scanned with a spatial resolution of 80 μm (see Methods for detailed explanation of imaging procedure). To account for inter-run variability and intra-run signal drift, we ablated laboratory-prepared homogenous reference materials^18^ at the start and end of each sample scan, and the mean calculated counts per second per μg per g (CPS per μg per g) values were interpolated over the period of the image collection to provide an estimate of the calibration factor for each row of pixels (*i.e.* each laser scan line). Individual values in each row were then multiplied by the row's calibration factor (ESI Fig. 1†) to ensure pixel values, and thereby spatial metal concentrations, were comparable between samples. For our reference materials, the CPS per μg per g calibration coefficients for Fe, Cu and Zn were constant for the 18 day period over which experiments were performed (ESI Table 1†).

### Alignment of slices and creation of 3D voxelgram

Images were acquired using LA-ICP-MS according to methods we have previously described.^19^ Following background subtraction, segmentation and masking (see Methods), the data were structured as a stack of 2D images, with images rotated about a central axis (as depicted in Fig. 1). To perform image alignment, we implemented a pyramidal pixel registration technique that minimised the difference between a ‘reference’ image (the preceding section) and a ‘test’ image (the current section), the latter undergoing an affine transformation, *Q*, with parameter set P; minimised such that:*ε* ^2^ = ‖*f*_R_(*x*) – *Q*_P_{*f*_T_(*x*)}‖^2^where *f*_R_(*x*) and *f*_T_(*x*) (ref. 20) are 3rd order polynomial spline functions representing the reference and test images, respectively. No change in grey-scale, skew or shear was evaluated or applied, since the original concentration data and slice morphology were required after the transform to maintain the varying anatomical structure throughout the brain.

Image registration was performed across 4 levels, each down-sampled by a factor of 2. Down-sampling is a useful and more computationally-efficient method for preliminary registration with a large, mismatched dataset,^21^ as evidenced by the changing morphology of our consecutive brain sections. Following initial registration, the next level-up images (*i.e.* those less down-sampled) were aligned using the parameters of the previous level as a starting point for optimisation, eventually returning to the original image resolution with a fully registered dataset. For the first level of registration of the reference and test images, down-sampling was set to 1/16th of the original. This method reduced computational analysis time, as the most intensive step was the initial registration, which lacked estimates of transformation parameters. For subsequent levels, estimates of the transformation parameters calculated from the preceding level significantly reduced computational overheads such that upon the 4th level, only fine-scale orientation adjustments were required. This down-sampled alignment procedure also reduced the likelihood of settling upon a localised false minimum due to outliers or small-scale anomalies,^20^ such as random spikes (*i.e.* single data points significantly higher than surrounding pixels viewed in a 9 × 9 matrix) arising from aberrant particles or the electron multiplier of the ICP-MS.

The registration process may be performed across single or multiple elements with retrospective application of the optimal transformation parameters across all elements in the image stack. This approach provided flexibility in selection of contrasting data to optimise registration, where several measured elements are available for use as alignment features. For example, although alignment with ^31^P in brain tissue typically provided a high signal-to-noise ratio with respect to tissue ^31^P content *versus* low background signal intensity, it demonstrated few sharply defined features beyond demarcating tissue boundaries. In contrast, labelling of a specific cell type with an exogenous metal marker highlighted more distinct areas and structural features according to a specific biochemical feature, which could be used for more precise alignment.

**Fig. 2:**
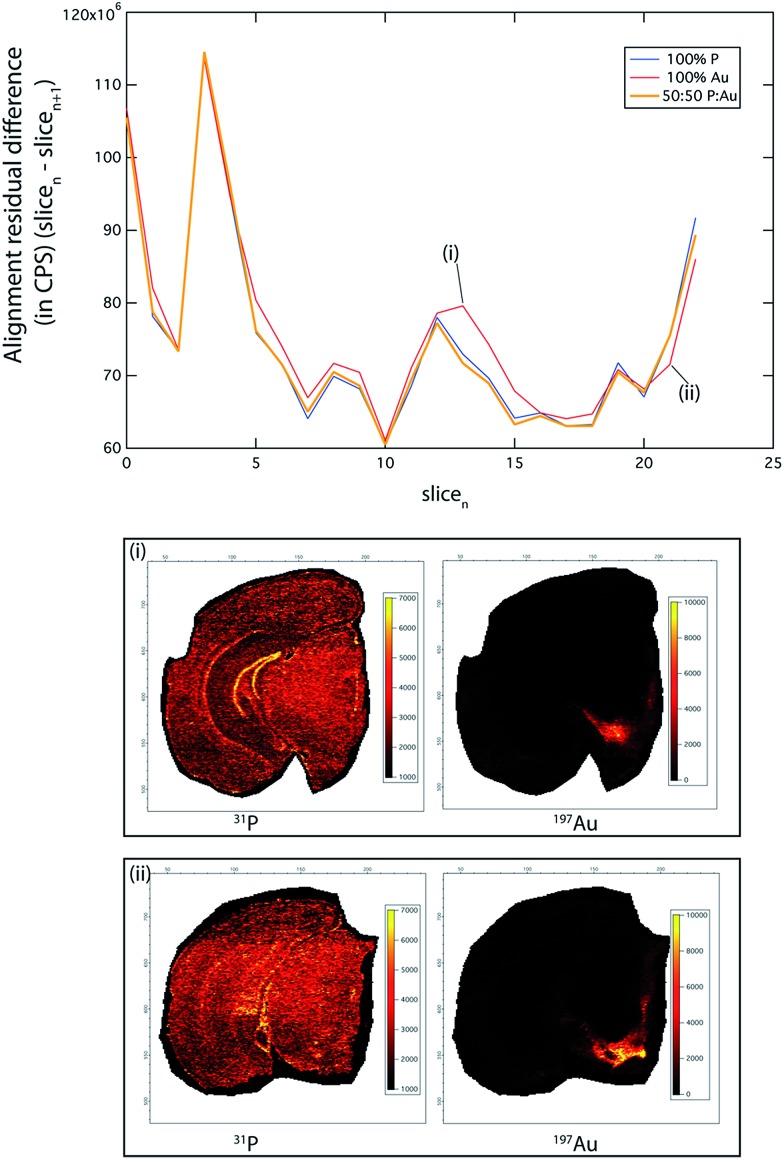
Consecutive image alignment. An alignment suitability comparison examining the pixel intensity difference between each slice and the following slice of a 3D image stack. The “100% P” line shows the difference between each slice when only P is used for the alignment parameter calculation, and similarly with using only the Au channel in the calculation: “100% Au”. The “50 : 50 P : Au” line shows the result of using a combined P and Au channel for alignment. Although P is ubiquitous within the brain, it only produces significantly better alignment than Au in areas where it defines sharply delineated features (dentate gyrus), such as shown in (i). Similarly, Au by itself produces the best alignment in parts of the brain where it defines high-intensity features (*e.g.* TH-rich SNc and VTA, shown in (ii)). Thus, using either of these channels will produce differing qualities of alignment depending on the slice location within the brain. In general, the combined P : Au channel produces the best overall alignment result, independent of the slice location within the brain.

To demonstrate this, we ablated 23 serial coronal sections at 90 μm intervals labelled with gold (Au) nanoparticle tagged tyrosine hydroxylase (TH) positive neurons (see Hare *et al.* ^12^ and Methods for immunolabelling protocols). This approximately 2 mm-thick segment of the murine brain (bregma –2.3 mm to –4.3 mm) encompassed the TH-rich substantia nigra pars compacta (SNc) and ventral tegmental area (VTA). We compared the accuracy of our image alignment approach using only the ^31^P signal, only the ^197^Au signal, and a combined channel of ^31^P and ^197^Au. To calculate the combined channel, the ^31^P and ^197^Au CPS signals were normalised to greyscale, with assigned values between 0 and 255. The value of each pixel in this new channel was calculated as 0.5 × ^31^P_N_ + 0.5 × ^197^Au_N_, where the N subscript represented the normalised image (Fig. 2).

The P_N_ + Au_N_ channel provided the most accurate alignment (total alignment residual difference ^31^P_N_ + ^197^Au_N_: 0.551; ^31^P only: 0.553; ^197^Au only: 0.566), due to clearly defined, high-intensity features across the whole image stack. Of note, the residual differences reported here reflect the sum of *all* differences between the corresponding pixels in the two images. While an alignment residual difference of 0.5 may appear to be high, the morphology between adjacent brain sections is variable. Thus, perfect alignment accuracy (*i.e.*, an alignment residual difference of 0) is not possible, and it is reasonable that around 50% of pixels returned similar values. Additionally, small levels of instrument drift may contribute to this variation.

Phosphorus was widely distributed within the mouse brain and was suitable for alignment of the whole image, with superior performance in areas of fine-scale detail, such as the dentate gyrus ((i) in Fig. 2). Similarly, where there was sufficient ^197^Au signal intensity to define sharp features (*i.e.* SNc and VTA; (ii) in Fig. 2), there was improved alignment. However, ^31^P performed as the better overall single channel, due to its ubiquitous distribution and comparatively homogenous distribution, whereas TH (as ^197^Au) is found only in discrete brain regions (see below).

### Data visualisation and multi-criteria voxelgram

Data visualisation in a multi-channel 3D environment is challenging, since there is a 3D data array produced for *each* channel (*i.e.*, a 3D image stack for ^56^Fe, ^63^Cu, ^66^Zn, *etc.*). In a 2D image, multiple channels may be displayed by assigning colour to up to three channels at a time, usually red, green and blue,^22,23^ with the intensity of the colour proportional to the concentration of that channel. Displaying three or more channels simultaneously in three-dimensions often results in obscuration of the inner voxels, which may be mitigated by making the outer voxels more transparent.^24^ While this is appropriate in some cases, modelling metal distribution in the brain may require comparison of the outer and inner voxels simultaneously. We used a multi-criteria voxelgram approach to represent the combined metal content of each voxel simultaneously. Histograms of voxel values for each channel displayed the concentration and distribution of each element.

For each channel, a portion of the histogram can be highlighted, and only voxels with values that fall within this region are visible in a second window showing a 3D representation (voxelgram) of the data. For example, for a voxel to be shown in our 3D voxelgram, it must have a value within the selected region for each channel (combined in a Boolean sense). To demonstrate how multi-criteria voxelgram imaging may be used to define specific brain regions, we manipulated the ranges of voxel values displayed to define the hippocampal formation (HIF; ESI Movie 1†).

**Fig. 3:**
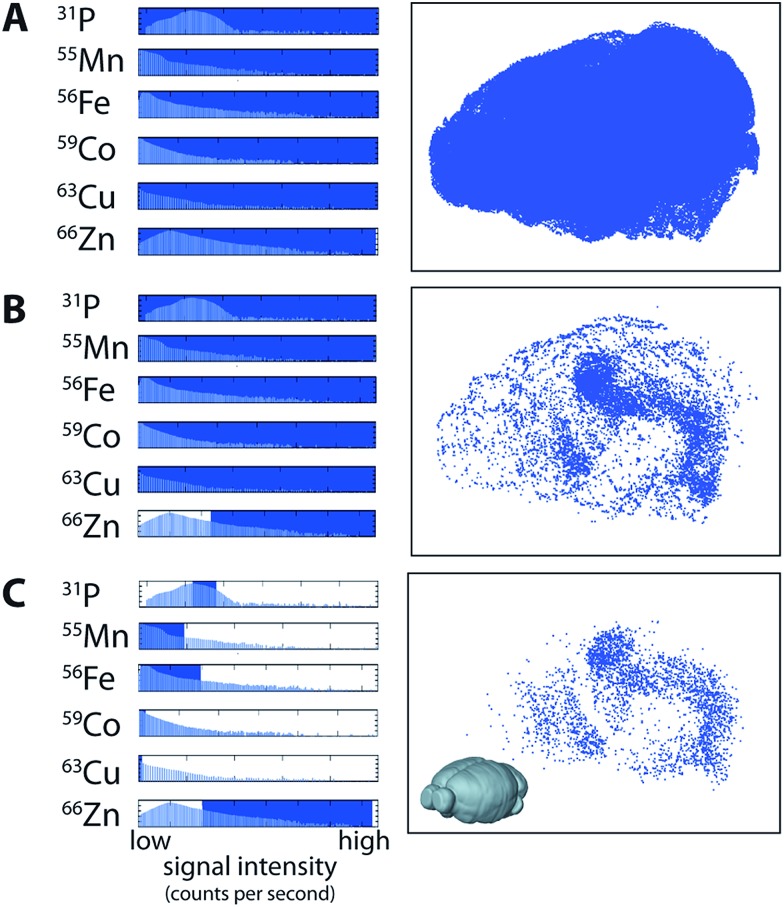
Multi-criteria voxelgrams of ^31^P, ^55^Mn, ^56^Fe, ^59^Co, ^63^Cu and ^66^Zn define the hippocampal formation. (a) Three-dimensional voxelgrams inclusive of all pixels containing spectra for each measured mass provides a complete rendering of brain tissue boundaries. By manipulating the portion of pixels displayed (determined by selecting from the histogram of pixel values; blue rectangles), areas corresponding to specific concentration ranges of elements of interest may be displayed, shown as red rectangles. Accordingly, areas of high Zn (>4.5 μg g^–1^; (b) were selected to show distinct regions corresponding to high concentrations in the cortex and hippocampal formation. These parameters were further manipulated to display voxels according to user-defined range (*e.g.* P: 60 000–80 000 counts per second; Mn: 0–190 counts per second; Fe: 2–38 μg g^–1^; Co: 0–0.01 μg g^–1^; Cu: 0–1 μg g^–1^; and Zn: >4 μg g^–1^; (c), which in this case primarily defines the hippocampal formation independent of voxels in the cortex.

Voxels representative of all areas of ablated tissue were displayed when all measured channels were selected (Fig. 3a). Displaying only voxels containing >4.5 μg g^–1^ of Zn demarcated both the HIF in the deep brain and the outer cortex (Fig. 3b). Restricting voxels further to those containing only high Zn, μg g^–1^ Fe, μg g^–1^ Cu, μg g^–1^ Co and <2000 CPS Mn (note that Mn was not quantified due to a lack of appropriate tissue standards) and the third-quartile range (6–8 × 10^4^ CPS; Fig. 3c) for P revealed only voxels within the HIF, removing obscuring voxels in the cortex that did not meet our set criteria. Similarly, voxels defined by a single criterion (*e.g.* ^66^Zn defining the HIF) may be displayed as a unique colour channel with an additional parameter (*e.g.* areas of high ^56^Fe) displayed simultaneously when exported to ParaView 4.3.1 (Kitware, Sandia National Labs and CSimSoft; ESI Movie 2†), an open-source software package used for three-dimensional data visualisation.

### Enzyme–metal co-localisation in three dimensions

Our previous nanoparticle protein tagging approach^12^ used a secondary IgG antibody labelled with Au nanoparticles to supplant fluorophore-tagged IgG secondary antibodies used in normal immunohistochemical (IHC) workflows. Using 2D bioimaging, we learned that the SNc region of the mesencephalon, which is the site of marked dopaminergic cell loss in Parkinson's disease (PD), demonstrated concomitantly high levels of Fe and TH (the rate-limiting enzyme involved in dopamine synthesis and a proxy for dopamine concentration^25^). We hypothesised that this chemical environment was susceptible to increased oxidative stress driven by the dopamine–Fe redox couple, and we showed that 6-hydroxdopamine lesioning altered this chemical balance (in favour of a toxic dopamine metabolite) and stimulated parkinsonian neurodegeneration with parallel Fe accumulation.

Whilst cell loss is most prolific in the PD SNc, degeneration also occurs in additional nuclei; we therefore hypothesised that 3D mapping of Fe and TH levels, and their co-localisation patterns, would identify additional brain regions that exhibit a similar chemical environment. We adapted our previous approach to directly label mouse anti-TH, this time with the MaxPar® ytterbium-173 (^173^Yb) product that links the rare-earth element to the primary antibody by a polymer and maleimide-induced disulfide reduction. This antibody was used to stain an entire mouse brain, which underwent LA-ICP-MS imaging to map both the dopaminergic system and corresponding Fe, Cu and Zn concentrations. Alignment was performed using ^31^P.

**Fig. 4:**
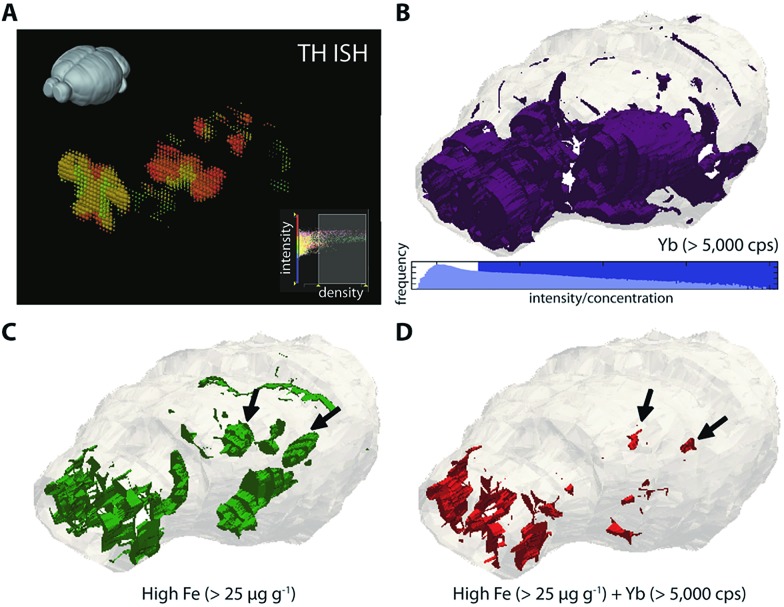
Three-dimensional co-localization of Fe and dopaminergic brain regions. (a) TH expression measured by ISH is available from the Allen Reference Atlas and Brain Explorer 2 platform. Comparing the 2–4th quartile ranges for both ISH data (density = 0.45–0.1 units) and ^173^Yb (>5000 counts per second; (b)) shows general agreement with TH expression in olfactory bulbs and mesencephalon. High Fe (>25 μg g^–1^; (c)) is also found in these regions, and colocalises with TH generally in the olfactory bulbs, and specifically in the substantia nigra pars compacta region (black arrows; (d)) of the midbrain.

To reconstruct 3D models of TH (and thereby dopamine, as ^173^Yb) distribution and its association with endogenous metals and compare against known TH expression patterns (from *in situ* hybridisation data of TH RNA, obtained from the open-access Allen Reference Atlas (ARA) and Brain Explorer 2 resources;^26^ Fig. 4a), smoothed voxelgrams generated in Biolite were exported to ParaView 4.3.1. In ParaView, 3D Delaunay triangulation was used to reconstruct 3D volume models of metal distribution according to the voxelgram output from Biolite (Fig. 4b–d; ESI Movie 3†). This movie demonstrated agreement with TH expression from the ARA, IHC profiling of mouse TH localisation,^27^ and also confirmed our previous findings related to co-localisation of TH and Fe in brain regions vulnerable to neurodegeneration (see Discussion).^12^

### Fuzzy clustering analysis

**Fig. 5:**
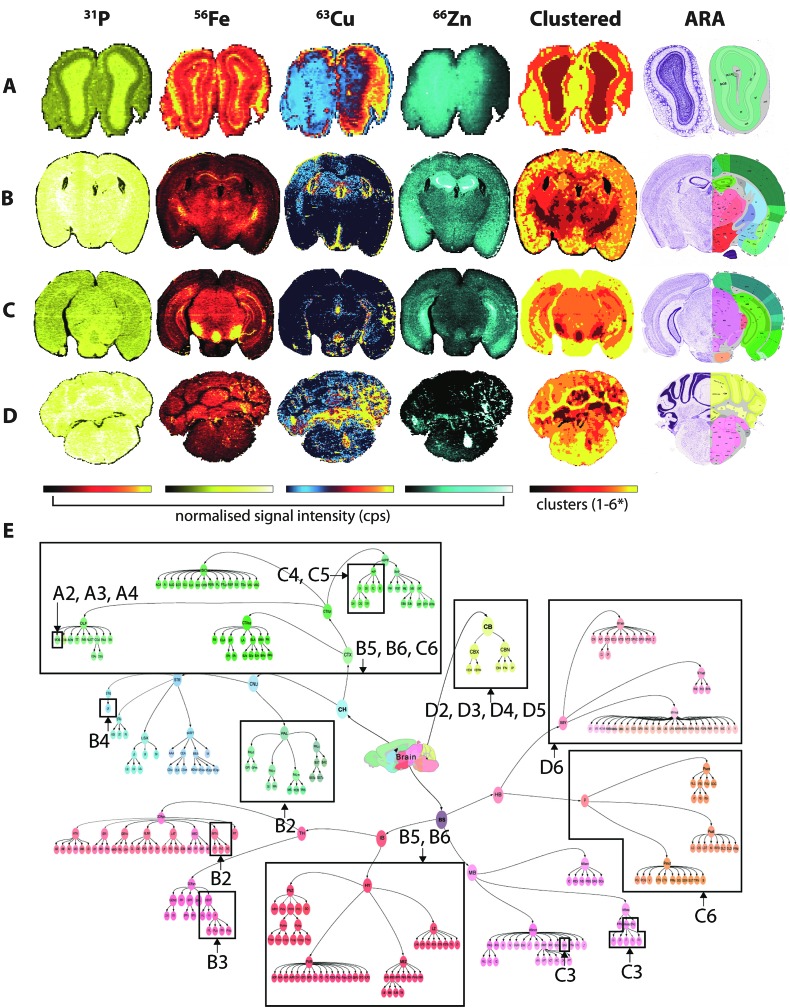
Fuzzy clustering in selected coronal sections. Normalised signal intensity for P, Fe, Cu and Zn distribution and resulting fuzzy clustering analysis was aligned with coronal sections from the ARA: bregma +4.27 mm (olfactory bulbs; A); –1.16 mm (thalamus; B); –3.58 mm (midbrain; C) and –6.36 mm (cerebellum; D). *4 clusters used for (a). Alignment of metal clusters with the brain structure hierarchical tree for the ARA for the 4 sections analysed could specifically identify nuclei classes, and in some cases, specific structures ((e) see Table 1).

Fine control of the visible voxels (as shown in Fig. 3 and 4) may be used to preselect specific metal concentrations, though the regions highlighted may not necessarily be indicative of significant data clusters representing anatomical brain regions. For example, to highlight the HIF, we manually set limits for voxel values, a process that may introduce user bias when attempting to define specific brain regions. As an alternative, we examined the five most representative elemental compositions within the brain based on the natural clustering patterns of the data, which identified anatomical brain regions that are considered similar with regard to metal content (Fig. 5).

The algorithm was based on *c*-means fuzzy clustering analysis^28,29^ previously used to characterise multiphase mineral assemblies. The clustering algorithm is iterative, and begins by randomly assigning a set number of cluster centres to the data. The membership of each data point (*i.e.* the inverse of the Euclidean distance to each cluster centre) is then calculated and the cluster centres are then re-calculated using the weighted average of memberships of each data point. Data points with greater membership provide additional weighting to the updated cluster centre values. This process iterates until there is little relative difference in the updated centre values, or a set number of iterations have been reached. Once the process is finished, the cluster centres may be reported, or each pixel/voxel may be assigned so that statistics for each cluster can be calculated.

This process is relatively straightforward to apply to a single data array. However, we have generalised the approach so that multiple channels from 2D and 3D datasets may be clustered. Each channel was converted to a 1D data array with each channel considered as a dimension for the cluster analysis. In this way, a full 3D dataset with *n* channels may be analysed to determine the natural clusters within the data. For each cluster, the average concentration (or normalised CPS) and range of values for each element was reported. These values may then be used in conjunction with the multi-criteria voxelgram to visualise the calculated clusters.

The 71 major structural/anatomical nuclei identified in the murine brain^30^ are not expected to be identifiable by metal concentration alone. We have previously reported that Fe, Cu and Zn display distributions generally in line with higher-level hierarchical ordering, with Fe highly concentrated in deep brain structures, Cu along the ventricular system and Zn in the hippocampus and outer cortex.^9^ Using fuzzy clustering analysis on 4 coronal sections chosen to reflect anterior-to-posterior progression through the mouse brain, we were able to subdivide brain regions further according to structures defined by the ARA (Table 1; ESI Table 2†). In some deep brain regions, structures were defined at the 3rd and 4th level of the ARA hierarchical tree (Fig. 5e), including the substantia nigra pars reticulata (SNr), midbrain raphe nuclei (RAmb) and caudoputamen (CP), while subdivisions of other higher order structures, such as the isocortex, were generally clustered into a single unit, reflecting their similar structural and chemical features, as opposed to functional roles.

**Table 1:** Hierarchical structure identification according to fuzzy clustering of metal distribution. CB = cerebellum; CP = caudoputamen; cpd = cerebral peduncle; CTX = cerebral cortex; HIP = hippocampal formation; HY = hypothalamus; IPN = interpeduncular nucleus; MOB = main olfactory bulb; MTN = midline groups of the dorsal thalamus; MY = medulla; P = pons; PAL = pallidum; RAmb = midbrain raphe nuclei SNc = substantia nigra pars compacta; SNr = substantia nigra pars reticulata; VENT = ventral part of the dorsal thalamus. n/a = not applicable, background single only. * For the section at bregma +4.27 mm only 4 clusters were discernable

Bregma ± (mm)	1		2		3		4		5		6	
	ARA classification	% image	ARA classification	% image	ARA classification	% image	ARA classification	% image	ARA classification	% image	ARA classification	% image
(A) +4.27 (olfactory bulbs)*	n/a	14	Fiber tracts, MOB	25	MOB	29	MOB	31	—	0	—	0
(B) –1.16 (thalamus)	n/a	6	MTN; PAL	10	VENT	17	CP	19	CTX; HY	22	CTX; HY	26
(C) –3.58 (midbrain)	n/a	0	—	0	RAmb; SNr	3	HIP; fiber tracts; SNc	11	HIP	40	CTX; P	46
(D) –6.36 (cerebellum)	n/a	2	CB	12	CB	15	CB	20	CB	25	Fiber tracts; MY	27

## Discussion

Relating metals to their neurological functions is a challenging task. Beyond just enzymes, metals are believed to interact with up to one-third of the known proteome.^1,31^ We recently made a conservative estimate that approximately 6600 human genes encode for metalloproteins,^32^ and a significant portion of these proteins are yet to have their appropriate metal cofactors assigned. Additionally, the ubiquity of metals throughout the brain presents a complex problem for discerning their functions in specific brain regions. While the roles of certain metals in the brain have been reasonably well defined, little is known the roles of other metals: for instance, it is known that Zn is found at high levels in the hippocampal region and is essential for memory function,^33^ though the reasons why Cu accumulates in subventricular zones in astrocytes, and increases with age, are unclear.^34,35^ The protocol described here provides a dual-faceted approach for decoding how metals relate to both neurological functions and neuroanatomy.

Metal metabolism is typically intertwined, and the implementation of our protocol may result in novel insights into the complex interactions among metals in different neurological conditions. For instance, neuronal Fe export is reliant upon the highly-abundant multi-copper oxidase ceruloplasmin,^36^ and genetic ablation of the ceruloplasmin gene in mice stimulates neuronal Fe overload and concurrent parkinsonism.^37^ Using our protocol, we would be able to identify voxels corresponding to reduced Cu and elevated Fe levels to produce a brain-wide picture of the effects of ceruloplasmin deficiency, and we could explore whether these effects are confined to vulnerable brain regions or are a system-wide phenomenon.

Recent advances using exogenous metal tagging of specific biomolecules enable the second, and perhaps most versatile, application of three-dimensional imaging of metals by LA-ICP-MS. Immunohistochemistry using rare-earth-tagged antibodies avoids many of the problems associated with fluorescence, such as intensity loss with time, staining artefacts and autofluorescence,^38^ and detection of metal-tagged antibodies using LA-ICP-MS is very sensitive, with detection limits as low as 0.1 ng.^39^ Here, we used anti-TH antibodies tagged with ^173^Yb to reconstruct the dopaminergic system according to TH distribution. The resulting map was in accordance with ISH data on TH RNA levels from the ARA^26^—and compared to the ARA, which reports ISH data at a 200 μm spatial resolution, our method not only is at a higher spatial resolution, but also more sensitive and specific.

Importantly, metal tagging of biomolecules enables the assessment of co-localisation of proteins with endogenous metals, which offers a highly-sensitive means for studying the interactions between proteins and metal cofactors. We previously used this technique to examine the 2D relationship between TH (as a proxy for dopamine) and Fe levels in a mouse model of Parkinson's disease: the autooxidation of dopamine is a potent source of reactive oxygen species in Parkinson's disease and is promoted in the presence of Fe,^40^ but prior to our study, little was known about the regional distributions of Fe in the parkinsonian brain. Confirming our previous report,^12^ we found that high Fe and high dopamine levels co-localised in the substantia nigra pars compacta (SNc; volume approx. 600 μm^3^) region (Fig. 4d), which was well defined from the Fe-rich yet dopamine-absent adjacent SNr region. Here, we found additional evidence for the co-localisation of dopamine and Fe in the hypothalamus, to which SNc dopaminergic neurons project and in which dysfunction is associated with non-motor symptoms of Parkinson's disease.^41^ Dopamine and iron also co-localised in the olfactory bulbs, which are particularly susceptible to 6-hydroxydopamine toxicity^42^ and in which dysfunction is thought to pre-date the onset of motor symptoms in Parkinson's disease.^43^ These—and only these—three regions showed evidence of co-localisation of this potent redox couple. Vulnerability may be due to increased oxidative stress arising from Fe-mediated dopamine auto-oxidation, which we previously observed in the SNc,^12^ and which is accentuated by administration of this neurotoxic breakdown product of the essential catecholamine.^44^ Further study using this protocol is a priority to fully characterise Fe-mediated vulnerability throughout the dopaminergic system.

It is important to note that our sample preparation methods are not exempt from possible post-mortem artefacts, though we took care to avoid any potential loss or contamination of metals. We previously reported that chemical fixation and cyroprotection techniques necessary to maintain tissue integrity influence metal levels, with transition metals losing around 25% of their total concentrations,^45^ although these metals still show distinct compartmentalisation that can be correlated with specific brain nuclei. We have also observed that the Fe content of deep brain structures like the midbrain does not significantly differ from unfixed frozen tissue taken from the same animal.^12^ This suggests that metal loss during fixation may be related to the amount of soluble or ionic material, though interestingly Zn concentrations remained highest in the HIF, where large amounts of soluble Zn^2+^ play an excitatory role.^46^

Numerous alignment strategies are available for image registration, though in examples like tomography, the process is somewhat simplified since offset and rotation are negligible factors. However, these factors must be taken into account when aligning cut tissue sections, particularly in a structurally-diverse organ such as the brain. A common alignment strategy for cut tissue sections is the ‘three landmarks’ approach, which is quite effective when comparing the same sample under different conditions, but less effective when landmarks are sparse. In our case, there is often limited commonality between slices of murine brain tissue, which contains a complex hierarchical structure of 71 major brain regions with multiple subregions,^30^ many of which have varying metal concentrations.^9^ This approach is also limited to the accuracy with which the three landmarks are chosen, with any error propagated to the resulting alignment. The pyramidal affine transformation approach described here is a more accurate method for image alignment, as it takes every pixel into account.

The Allen Reference Atlas, including genome-wide expression detail,^26^ has since expanded to include the developing mouse brain, mouse spinal cord, mouse brain connectome,^47^ human brain transcriptome,^48^ and most recently, the transcriptional features of the foetal human brain.^49^ Functional reference atlases describing genomic, transcriptomic and neural connectivity complement traditional anatomical atlases, and with interactive online resources and applications such as the Brain Explorer tool, relating structure to function is becoming more accessible to scientists. However, generation of large data sets, the need for high throughput histology and microscopy, and automated image analysis are all high-cost endeavours, which the ARA has benefited from a ‘venture philanthropy’ model.^6^ The outcomes from the ARA's open-access model have stimulated significant advances in the neurosciences, and this will only be supplemented further as more scientists continue to disseminate their research. The work described in this manuscript lays the groundwork for the development of the aforementioned ‘standard model’ of metal distributions and concentrations in the laboratory mouse brain.

## Conclusions

Imaging using LA-ICP-MS is evolving beyond simply providing two-dimensional recreations of metal distribution in single tissue sections. By employing antibody labelling with exogenous metal tagging, new potential for highly sensitive assessment of metal and protein co-localisation, which can provide unique information regarding the specific function of a metal or antigen, in either normal health or disease. The resources described here are another step towards the creation of a true metallomic atlas of the standard laboratory mouse, and sits at the interface of neurobiology and analytical chemistry.

## Methods and materials

### Animals

All animal experiments conformed to the Australian National Health and Medical Research Council standards of animal care and were carried out in accordance with the requirements of the Howard Florey Animal Ethics Committee. 16 month old male C57BL/6 mice were raised according to standard animal care protocols and fed normal chow and water *ad libitum*. Animals were killed with an overdose of sodium pentobarbitone (100 mg kg^–1^) and perfused with 30 mL of warmed (37 °C) 0.1 M phosphate buffered saline (PBS), pH 7.4. Tissue was briefly fixed in 4% paraformaldehyde in PBS until the brains sank, after which they were immersed overnight in two changes of 30% sucrose in PBS.^45^ Tissue was then frozen at –80 °C and mounted in O.C.T.™ *via* the medulla oblongata and upper spinal cord. After equilibrating at –20 °C, the brains were sectioned using PTFE-coated cryotome blades to 30 μm thickness at 90 μm intervals and mounted on standard microscope slides.

### LA-ICP-MS imaging

Imaging we performed using two LA-ICP-MS systems. The first consisted of a New Wave Research UP213 (Kennelec Scientific) Nd:YAG laser system with a two-volume large format cell connected to an Agilent 7500 cs ICP-MS (Agilent Technologies). The second was a New Wave Research NWR193 (Kennelec Scientific) ArF excimer laser with an Agilent 7700x ICP-MS. Both ICP-MS instruments were fitted with ‘s’ lenses for enhanced sensitivity. Argon was used as the carrier gas and laser fluence was set at 0.3 J cm^–2^ for all experiments. An 80 μm (50 μm for TH experiments) laser beam diameter was traversed across the sample at 320 μm s^–1^, with ICP-MS dwell times set according to the parameters outlined by Lear *et al.* ^50^ Hydrogen was used as a reaction gas in all experiments to reduce polyatomic interference on ^56^Fe by ^40^Ar^16^O.^51^ Hydrogen effectively removes ^40^Ar^16^O without reducing overall signal intensity, which is necessary to obtain adequate detection limits, as would be the case using a more indiscriminate collision gas like helium.

### IHC protocols – Au nanoparticle tagging for imaging alignment

IHC protocols to labelled TH-positive neurons was adapted from our previously described methods.^12^ Briefly, sections were air dried at room temperature and post-fixed in 2% (w/v) paraformaldehyde in phosphate buffered saline (PBS) pH 7.4 for 5 minutes. Sections were the rinsed 3 times in PBS before blocking for 30 min at room temperature in a buffer comprising 1% bovine serum albumin (BSA; Sigma, Sydney, Australia), 0.3% Triton X-100, 3% (v/v) normal goat serum (Sigma) in PBS. Sections were then incubated with a 1 : 3000 dilution of polyclonal anti-TH (Merk-Millipore) overnight at 4 °C. After washing in PBS, the samples were further incubated for 3 hours at room temperature with 1 : 100 goat anti-rabbit IgG with 10 nm Au nanoparticles preabsorbed (Abcam). Sections were again washed in PBS and covered with a silver enhancement solution (BBI International) to visualise the successful labelling of the TH neurons. Sections were then washed in deionised water and allowed to dry at room temperature before analysis.

### IHC protocols – Yb labelling for TH–Fe co-localisation

Carrier free sheep anti-mouse tyrosine hydroxlase (TH) polyclonal antibody (AF7566) was obtained from R&D Systems (Minneapolis, MN). The antibody (100 μg) was labelled with ytterbium-173 using the Yb-MaxPar® antibody conjugation kit (Fluidigm; formerly DVS Sciences, San Francisco, CA) according to the manufacturer's directions. The concentration of the resulting antibody was estimated by absorbance at 280 nm. Following blocking as described above, the sections were incubated for 16 hours at room temperature with ^173^Yb anti-TH antibody diluted 1 : 1000 in the same buffer. After this time, sections were rinsed 3 × 5 min in PBS and 1 × 5 min in 18 MΩ water. Sections were air dried and stored at room temperature until analysis. ^137^Y-labeled isotype controls are shown in ESI Fig. 2.†

### Initial data preparation and background removal

Biolite provides an interactive user interface with a clear workflow for image alignment. The initial data preparation stage was to import data from the mass spectrometer, in comma separated value files (.csv). The data was arranged as rows in a two-dimensional image of the entire scanned area, with each pixel representing a mass spectrometric datum, with a separate image for each measured mass-to-charge (*m*/*z*) value (*e.g.* ^56^Fe, ^63^Cu, ^66^Zn *etc.*).

Several regions were selected that represented the background (ESI Fig. 3†) using an appropriate channel, typically the *m*/*z* with the highest signal-to-noise ratio (in this case ^31^P; ESI Fig. 4a and b†). Background was removed by creating a surface based on an interpolation between each background region to create an image of the background counts, using the built-in Igor Pro function *ImageRemoveBackground* (ESI Fig. 4c†).^17^ This background image for each measured *m*/*z* was then subtracted from the original image to create a background-subtracted image for all measured channels (ESI Fig. 4d†). This approach compensated for long-term, non-linear instrument drift (ESI Fig. 4e†) that may have occurred over extended runtimes (often over 24 hours), and was effective in near-complete removal of all background signal over the course of a single imaging experiment (ESI Fig. 4f†).

### Signal normalisation and quantification

Background-subtracted images are typically normalised to standards in order to correct for sensitivity drift in the ICP-MS, and to provide spatial quantification of the concentration of the analysed material. In our case, reference materials were analysed at the start and end of each experimental batch to calculate the calibration factor for each measured mass (ESI Fig. 5†). Considerations normally a concern using spot analysis (such as the downhole ‘wall effect’) were less of a concern when ablating thin tissue sections. Here, rastering does not produce a downhole effect, and we are effectively only comparing the surface of our sample against the surface of out reference materials, which are matrix-matched and cut to an equivalent thickness. Variations in transport efficiency (or any other effect) would affect both standard and sample, and thus this would be exclusive.

### Section segmentation and background masking

Following signal normalisation, the order (anterior to posterior) and spatial extent of each slice was defined (ESI Fig. 6a†). The exact dimensions of each slice were not critical, as each background was further masked by manually outlining the tissue boundaries (ESI Fig. 6b†), which may include nearby or overlapping sections. A polygon-drawing tool was employed to enclose each individual section to avoid interference from background anomalies or nearby sections during the alignment process.

## References

[cit1] Waldron K. J., Rutherford J. C., Ford D., Robinson N. J. (2009). Nature.

[cit2] Raichle M. E., Gusnard D. A. (2002). Proc. Natl. Acad. Sci. U. S. A..

[cit3] Que E. L., Domaille D. W., Chang C. J. (2008). Chem. Rev..

[cit4] Barnham K. J., Bush A. I. (2014). Chem. Soc. Rev..

[cit5] Paxinos G. and Watson C., The Rat Brain in Stereotaxic Coordinates, Academic Press, San Diego, 1982.

[cit6] Jones A. R., Overly C. C., Sunkin S. M. (2009). Nat. Rev. Neurosci..

[cit7] McRae R., Bagchi P., Sumalekshmy S., Fahrni C. J. (2009). Chem. Rev..

[cit8] Miller L. M., Wang Q., Telivala T. P., Smith R. J., Lanzirotti A., Miklossy J. (2006). J. Struct. Biol..

[cit9] Hare D. J., Lee J. K., Beavis A. D., van Gramberg A., George J., Adlard P. A., Finkelstein D. I., Doble P. A. (2012). Anal. Chem..

[cit10] Matusch A., Fenn L. S., Depboylu C., Klietz M., Strohmer S., McLean J. A., Becker J. S. (2012). Anal. Chem..

[cit11] Hutchinson R. W., Cox A. G., McLeod C. W., Marshall P. S., Harper A., Dawson E. L., Howlett D. R. (2005). Anal. Biochem..

[cit12] Hare D. J., Lei P., Ayton S., Roberts B. R., Grimm R., George J. L., Bishop D. P., Beavis A. D., Donovan S. J., McColl G., Volitakis I., Masters C. L., Adlard P. A., Cherny R. A., Bush A. I., Finkelstein D. I., Doble P. A. (2014). Chem. Sci..

[cit13] Hare D. J., George J. L., Grimm R., Wilkins S., Adlard P. A., Cherny R. A., Bush A. I., Finkelstein D. I., Doble P. (2010). Metallomics.

[cit14] Berry R. P., Ibbotson M. R. (2010). PLoS One.

[cit15] Schormann T., von Matthey M., Dabringhaus A., Zilles K. (1993). Bioimaging.

[cit16] de Jonge M. D., Holzner C., Baines S. B., Twining B. S., Ignatyev K., Diaz J., Howard D. L., Legnini D., Miceli A., McNulty I., Jacobsen C. J., Vogt S. (2010). Proc. Natl. Acad. Sci. U. S. A..

[cit17] Paton C., Hellstrom J., Paul B., Woodhead J., Hergt J. (2011). J. Anal. At. Spectrom..

[cit18] Hare D. J., Lear J., Bishop D., Beavis A., Doble P. A. (2013). Anal. Methods.

[cit19] Hare D., Reedy B., Grimm R., Wilkins S., Volitakis I., George J., Cherny R. A., Bush A. I., Finkelstein D. I., Doble P. (2009). Metallomics.

[cit20] Thévenaz P., Ruttimann U. E., Unser M. (1998). IEEE Trans. Image Process..

[cit21] Zhilkin P., Alexander M. E. (2000). Magn. Reson. Imaging.

[cit22] Angelo M., Bendall S. C., Finck R., Hale M. B., Hitzman C. (2014). Nat. Med..

[cit23] Giesen C., Wang H. A. O., Schapiro D., Zivanovic N., Jacobs A., Hattendorf B., Schüffler P. J., Grolimund D., Buhmann J. M., Brandt S., Varga Z., Wild P. J., Günther D., Bodenmiller B. (2014). Nat. Methods.

[cit24] Pfister H., Lorensen B., Bajaj C., Kindlmann G., Schroeder W., Avila L. S., Raghu K. M., Machiraju R., Lee J. (2001). IEEE Computer Graphics and Applications.

[cit25] Bacopoulos N. G., Bhatnagar R. K. (1977). J. Neurochem..

[cit26] Lein E. S., Hawrylycz M. J., Ao N., Ayres M., Bensinger A., Bernard A., Boe A. F., Boguski M. S., Brockway K. S., Byrnes E. J., Chen L., Chen L., Chen T.-M., Chi Chin M., Chong J., Crook B. E., Czaplinska A., Dang C. N., Datta S., Dee N. R., Desaki A. L., Desta T., Diep E., Dolbeare T. A., Donelan M. J., Dong H.-W., Dougherty J. G., Duncan B. J., Ebbert A. J., Eichele G., Estin L. K., Faber C., Facer B. A., Fields R., Fischer S. R., Fliss T. P., Frensley C., Gates S. N., Glattfelder K. J., Halverson K. R., Hart M. R., Hohmann J. G., Howell M. P., Jeung D. P., Johnson R. A., Karr P. T., Kawal R., Kidney J. M., Knapik R. H., Kuan C. L., Lake J. H., Laramee A. R., Larsen K. D., Lau C., Lemon T. A., Liang A. J., Liu Y., Luong L. T., Michaels J., Morgan J. J., Morgan R. J., Mortrud M. T., Mosqueda N. F., Ng L. L., Ng R., Orta G. J., Overly C. C., Pak T. H., Parry S. E., Pathak S. D., Pearson O. C., Puchalski R. B., Riley Z. L., Rockett H. R., Rowland S. A., Royall J. J., Ruiz M. J., Sarno N. R., Schaffnit K., Shapovalova N. V., Sivisay T., Slaughterbeck C. R., Smith S. C., Smith K. A., Smith B. I., Sodt A. J., Stewart N. N., Stumpf K.-R., Sunkin S. M., Sutram M., Tam A., Teemer C. D., Thaller C., Thompson C. L., Varnam L. R., Visel A., Whitlock R. M., Wohnoutka P. E., Wolkey C. K., Wong V. Y., Wood M., Yaylaoglu M. B., Young R. C., Youngstrom B. L., Feng Yuan X., Zhang B., Zwingman T. A., Jones A. R. (2007). Nature.

[cit27] Zeiss C. J. (2005). Vet. Pathol..

[cit28] Bezdek J. C., Pattern recognition with fuzzy objective function algorithms, Plenum Press, New York, 1981.

[cit29] Paul B., Woodhead J. D., Paton C., Hergt J. M., Hellstrom J., Ashley Norris C. (2014). Geostand. Geoanal. Res..

[cit30] Dong H. W., The Allen reference atlas: a digital color brain atlas of the C57Bl/6J male mouse, John Wiley & Sons, Hoboken, 2008.

[cit31] Waldron K. J., Robinson N. J. (2009). Nat. Rev. Microbiol..

[cit32] Lothian A., Hare D. J., Grimm R., Ryan T. M., Masters C. L., Roberts B. R. (2013). Front. Aging Neurosci..

[cit33] Sindreu C., Palmiter R. D., Storm D. R. (2011). Proc. Natl. Acad. Sci. U. S. A..

[cit34] Scheiber I. F., Mercer J. F. B., Dringen R. (2014). Prog. Neurobiol..

[cit35] Pushkar Y., Robison G., Sullivan B., Fu S. X., Kohne M., Jiang W., Rohr S., Lai B., Marcus M. A., Zakharova T., Zheng W. (2013). Aging Cell.

[cit36] Moos T., Nielsen T. R., Skjørringe T., Morgan E. H. (2007). J. Neurochem..

[cit37] Ayton S., Lei P., Duce J. A., Wong B. X. W., Sedjahtera A., Adlard P. A., Bush A. I., Finkelstein D. I. (2013). Ann. Neurol..

[cit38] Wilson S. M., Bacic A. (2012). Nat. Protoc..

[cit39] Asai S., Limbeck A. (2015). Talanta.

[cit40] Linert W., Herlinger E., Jameson R. F., Kienzl E., Jellinger K., Youdim M. B. H. (1996). Biochim. Biophys. Acta, Mol. Basis Dis..

[cit41] Chaudhuri K. R., Schapira A. H. (2009). Lancet Neurol..

[cit42] Lazarini F., Gabellec M.-M., Moigneu C., de Chaumont F., Olivo-Marin J.-C., Lledo P.-M. (2014). J. Neurosci..

[cit43] Ross G. W., Petrovitch H., Abbott R. D., Tanner C. M., Popper J., Masaki K., Launer L., White L. R. (2008). Ann. Neurol..

[cit44] Hare D. J., Adlard P. A., Doble P. A., Finkelstein D. I. (2013). Metallomics.

[cit45] Hare D. J., George J. L., Bray L., Volitakis I., Vais A., Ryan T. M., Cherny R. A., Bush A. I., Masters C. L., Adlard P. A., Doble P. A., Finkelstein D. I. (2014). J. Anal. At. Spectrom..

[cit46] Assaf S. Y., Chung S.-H. (1984). Nature.

[cit47] Oh S. W., Harris J. A., Ng L., Winslow B., Cain N., Mihalas S., Wang Q., Lau C., Kuan L., Henry A. M., Mortrud M. T., Ouellette B., Nguyen T. N., Sorensen S. A., Slaughterbeck C. R., Wakeman W., Li Y., Feng D., Ho A., Nicholas E., Hirokawa K. E., Bohn P., Joines K. M., Peng H., Hawrylycz M. J., Phillips J. W., Hohmann J. G., Wohnoutka P., Gerfen C. R., Koch C., Bernard A., Dang C., Jones A. R., Zeng H. (2014). Nature.

[cit48] Hawrylycz M. J., Lein E. S., Guillozet-Bongaarts A. L., Shen E. H., Ng L., Miller J. A., van de Lagemaat L. N., Smith K. A., Ebbert A., Riley Z. L., Abajian C., Beckmann C. F., Bernard A., Bertagnolli D., Boe A. F., Cartagena P. M., Chakravarty M. M., Chapin M., Chong J., Dalley R. A., Daly B. D., Dang C., Datta S., Dee N., Dolbeare T. A., Faber V., Feng D., Fowler D. R., Goldy J., Gregor B. W., Haradon Z., Haynor D. R., Hohmann J. G., Horvath S., Howard R. E., Jeromin A., Jochim J. M., Kinnunen M., Lau C., Lazarz E. T., Lee C., Lemon T. A., Li L., Li Y., Morris J. A., Overly C. C., Parker P. D., Parry S. E., Reding M., Royall J. J., Schulkin J., Sequeira P. A., Slaughterbeck C. R., Smith S. C., Sodt A. J., Sunkin S. M., Swanson B. E., Vawter M. P., Williams D., Wohnoutka P., Zielke H. R., Geschwind D. H., Hof P. R., Smith S. M., Koch C., Grant S. G. N., Jones A. R. (2012). Nature.

[cit49] Miller J. A., Ding S.-L., Sunkin S. M., Smith K. A., Ng L., Szafer A., Ebbert A., Riley Z. L., Royall J. J., Aiona K., Arnold J. M., Bennet C., Bertagnolli D., Brouner K., Butler S., Caldejon S., Carey A., Cuhaciyan C., Dalley R. A., Dee N., Dolbeare T. A., Facer B. A. C., Feng D., Fliss T. P., Gee G., Goldy J., Gourley L., Gregor B. W., Gu G., Howard R. E., Jochim J. M., Kuan C. L., Lau C., Lee C.-K., Lee F., Lemon T. A., Lesnar P., McMurray B., Mastan N., Mosqueda N., Naluai-Cecchini T., Ngo N.-K., Nyhus J., Oldre A., Olson E., Parente J., Parker P. D., Parry S. E., Stevens A., Pletikos M., Reding M., Roll K., Sandman D., Sarreal M., Shapouri S., Shapovalova N. V., Shen E. H., Sjoquist N., Slaughterbeck C. R., Smith M., Sodt A. J., Williams D., Zöllei L., Fischl B., Gerstein M. B., Geschwind D. H., Glass I. A., Hawrylycz M. J., Hevner R. F., Huang H., Jones A. R., Knowles J. A., Levitt P., Phillips J. W., Šestan N., Wohnoutka P., Dang C., Bernard A., Hohmann J. G., Lein E. S. (2014). Nature.

[cit50] Lear J., Hare D., Adlard P., Finkelstein D., Doble P. (2012). J. Anal. At. Spectrom..

[cit51] Lear J., Hare D. J., Fryer F., Adlard P. A., Finkelstein D. I., Doble P. A. (2012). Anal. Chem..

