# Correction: Visualising mouse neuroanatomy and function by metal distribution using laser ablation-inductively coupled plasma-mass spectrometry imaging

**DOI:** 10.1039/c6sc90060g

**Published:** 2016-09-07

**Authors:** Bence Paul, Dominic J. Hare, David P. Bishop, Chad Paton, Van Tran Nguyen, Nerida Cole, Megan M. Niedwiecki, Erica Andreozzi, Angela Vais, Jessica L. Billings, Lisa Bray, Ashley I. Bush, Gawain McColl, Blaine R. Roberts, Paul A. Adlard, David I. Finkelstein, John Hellstrom, Janet M. Hergt, Jon D. Woodhead, Philip A. Doble

**Affiliations:** a School of Earth Sciences , The University of Melbourne , Parkville , Victoria 3052 , Australia; b The Florey Institute of Neuroscience and Mental Health , The University of Melbourne , Parkville , 3052 , Victoria , Australia; c Elemental Bio-imaging Facility , University of Technology Sydney , Broadway , 2007 , New South Wales , Australia . Email: dominic.hare@uts.edu.au ; Tel: +61 2 9512 1792; d Senator Frank R. Lautenberg Environmental Health Sciences Laboratory , Department of Preventive Medicine , Icahn School of Medicine at Mount Sinai , New York , 10029 , New York , USA; e Centre for Star and Planet Formation , Geological Museum , University of Copenhagen , Øster Voldgade 5-7 , DK-1350 Copenhagen , Denmark

## Abstract

Correction for ‘Visualising mouse neuroanatomy and function by metal distribution using laser ablation-inductively coupled plasma-mass spectrometry imaging’ by Bence Paul *et al.*, *Chem. Sci.*, 2015, **6**, 5383–5393.



## 

A funder was omitted in the Acknowledgements section and the following text should have been added:

This work was also supported by the Ramaciotti Foundation.

The Royal Society of Chemistry apologises for these errors and any consequent inconvenience to authors and readers.

