# Supplementary material for: Visualising mouse neuroanatomy and function by metal distribution using laser ablation-inductively coupled plasma-mass spectrometry imaging
Source: Chem Sci. 2015 Jul 27;6(10):5383–93. doi: 10.1039/c5sc02231b (PMC5669312; doi:10.1039/c5sc02231b)
Supplement: Supplementary file 1 [file SC-006-C5SC02231B-s001.pdf]

Electronic Supplementary Material (ESI) for Chemical Science.
This journal is © The Royal Society of Chemistry 2015

Supplementary Information for:

Visualizing mouse neuroanatomy and function by metal distribution using laser ablation-inductively coupled plasma-mass spectrometry imaging

Bence Paul[1,2†], Dominic J. Hare[2,3,4†], David P. Bishop[3], Chad Paton[5], Van Tran Nguyen[3], Nerida Cole[3], Megan M. Niedwiecki[4], Erica Andreozzi[2], Angela Vais[2], Jessica L. Billings[2], Lisa Bray[2], Ashley I. Bush[2], Gawain McColl[2], Blaine R. Roberts[2], Paul A. Adlard[2], David I. Finkelstein[2], John Hellstrom[1], Janet M. Hergt,[1] Jon D. Woodhead[1] & Philip A. Doble[3*]*

[1] School of Earth Sciences, The University of Melbourne, Parkville, Victoria, 3052, Australia.

[2] The Florey Institute of Neuroscience and Mental Health, The University of Melbourne, Parkville, 3052, Victoria, Australia.

[3] Elemental Bio-imaging Facility, University of Technology Sydney, Broadway, 2007, New South Wales, Australia.

[4] Exposure Biology Laboratory, Lautenberg Environmental Health Sciences Laboratory, Department of Preventive Medicine, Icahn School of Medicine at Mount Sinai, New York, 10029, New York, United States of America.

[5] Centre for Star and Planet Formation, Geological Museum, University of Copenhagen, Øster Voldgade 5-7, DK-1350 Copenhagen, Denmark.

[†] Both authors contributed equally to this manuscript

Correspondence and requests for materials should be addressed to P.A.D. (email: philip.doble@uts.edu.au) or D.J.H (dominic.hare@uts.edu.au)

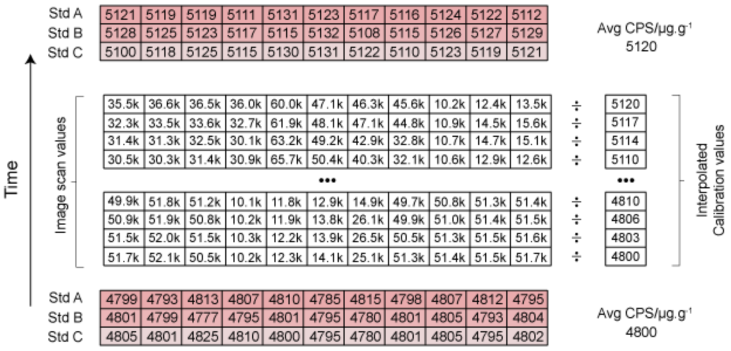

Supplementary Figure 1: Schematic of row-by-row interpolation of calibration factors (as CPS/μg g^{-1}) and division of row values by each row's interpolated factor. In this process, the average CPS/μg.g^{-1} for each in-house standard is calculated for each batch of standards (i.e. before and after each image). This average calibration value is interpolated to have the same number of points as there are rows in the image, and the values in each row are then divided by this interpolated value.

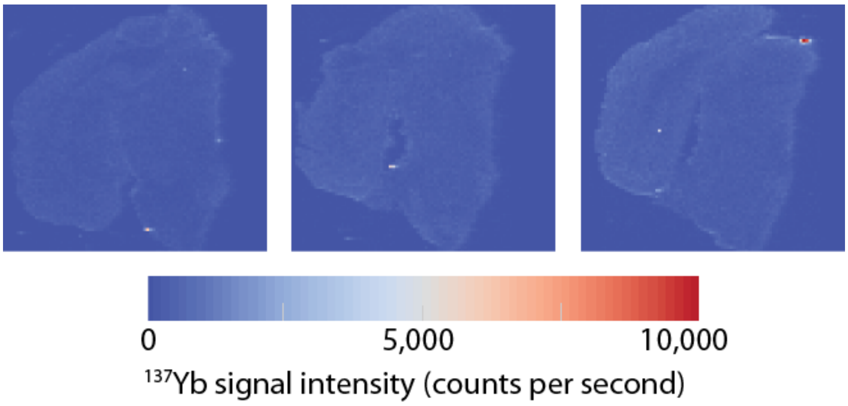

Supplementary Figure 2: Three replicate images of sections incubated with sheep IgG isotype controls labeled with Yb-173.

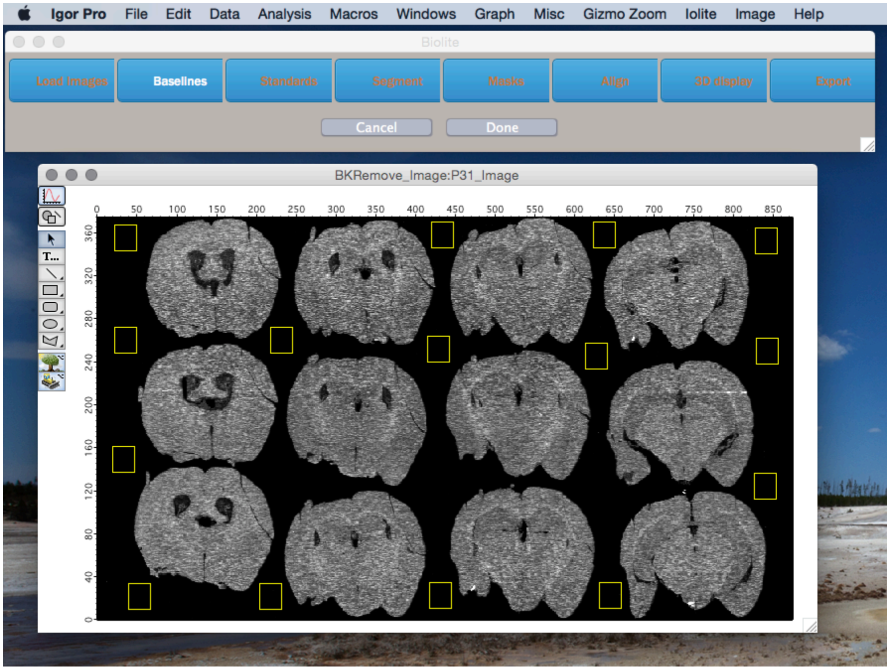

Supplementary Figure 3: Manual selection of background regions for surface interpolation.

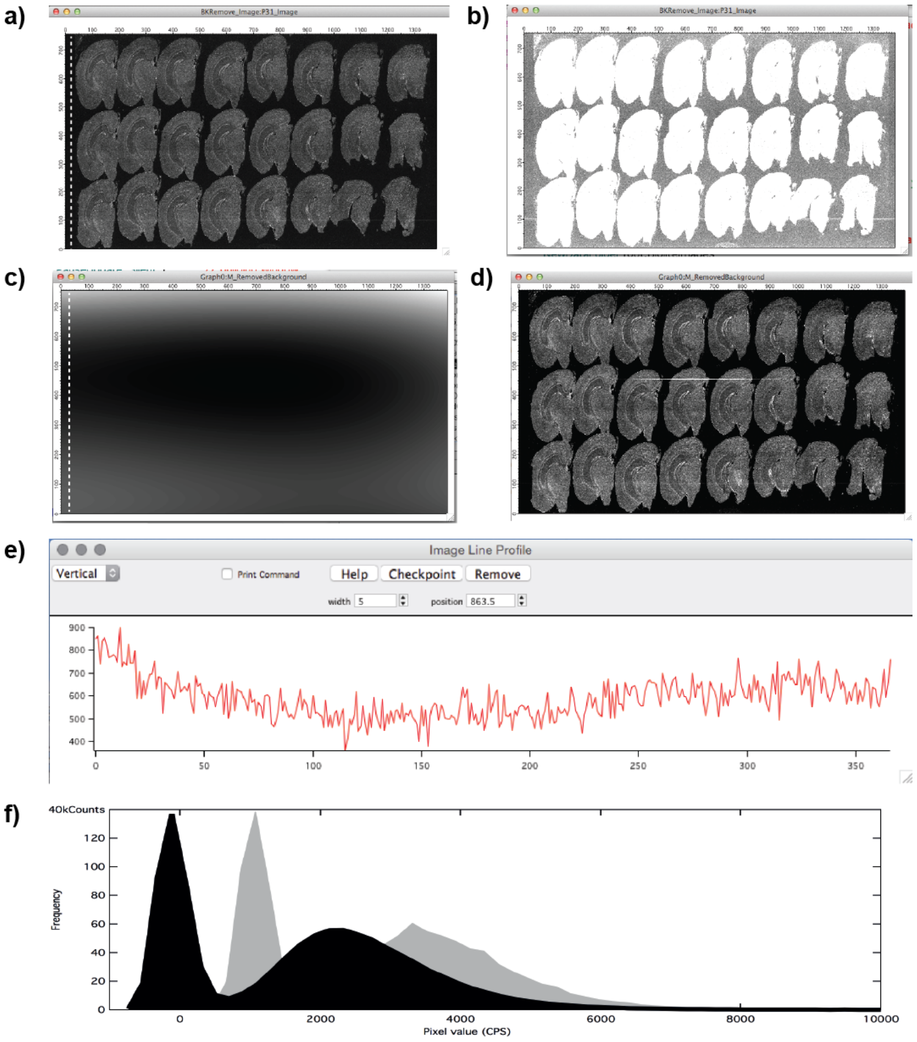

Supplementary Figure 4: Figure 4(a) shows the raw P31 channel image, before baseline correction. This same image is shown again in 4(b), but with the color-scale adjusted to show the backgrounds more clearly. From this image, it can be seen that the backgrounds are slightly higher (lighter grey) at the top and bottom of the image, compared to the backgrounds at the center (slightly darker grey). Figure 4(c) shows the result of interpolating between a series of areas selected as backgrounds to produce an estimate of the background variation. This image is subtracted on a pixel-by-pixel basis from (a) to produce the baseline-corrected image, (d). This approach corrects for the non-linear signal drift occurring over long instrument runtimes (in this case, the *y*-axis of the image; e), removing background signal whilst maintaining the distribution of pixels (f).

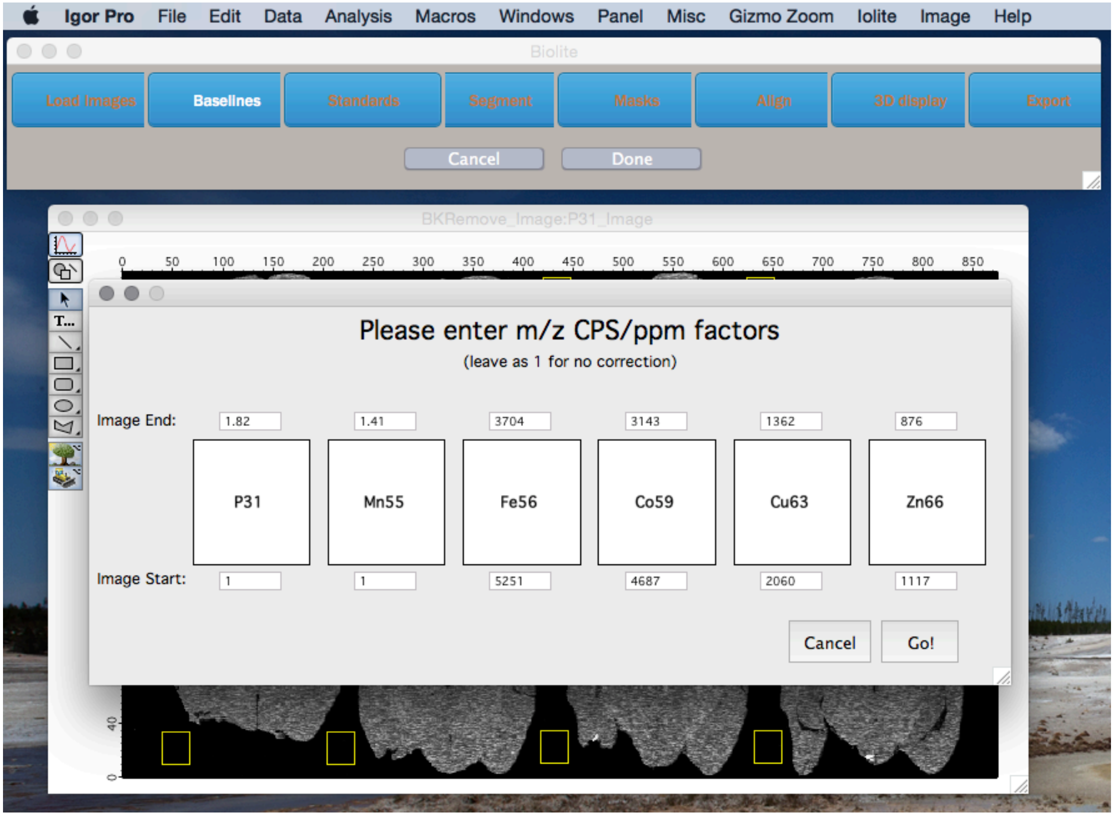

Supplementary Figure 5: Correction factor entry for each measured mass.

a)

b)

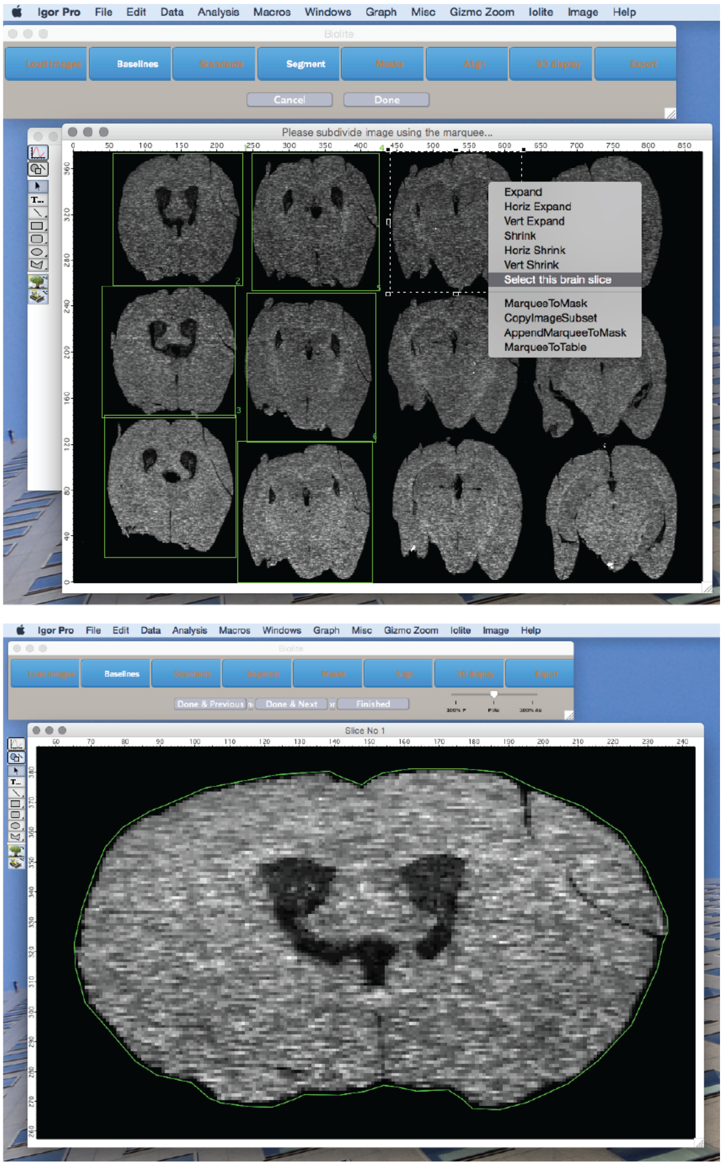

Supplementary Figure 6: a) Example of subdivision and slice ordering (1-12). b) Manual masking of tissue boundary.

Supplementary Table 1: Quantitative reproducibility for imaging experiments performed over an 18-day period, consisting of n = 32 measurements.

	**Fe**	**Cu**	**Zn**
**Average measured concentration (μg g^{-1})**	44	24	32
**Standard deviation**	4.5	3.0	3.5
**%RSD**	11	12	10
**Actual concentration (μg g^{-1})**	48 ± 1	24 ± 3	28 ± 2
**% accuracy**	-8	-2	15

Supplementary Table 2: Average values (cps; μg g^{-1} in italics) and 2 standard deviations for each cluster shown in **Fig. 4**. n/a = measured mass not used in clustering.

	**Cluster**	^{31}P		^{56}Fe		^{63}Cu		^{66}Zn	
**Bregma ± (mm)**		Average	2 SD	Average	2 SD	Average	2 SD	Average	2 SD
+ 4.27	1	10474	20924	*2.7141*	*5.9594*	n/a	n/a	n/a	n/a
	2	151460	18914	*23.088*	*9.8177*	n/a	n/a	n/a	n/a
	3	85799	14948	*18.957*	*12.101*	n/a	n/a	n/a	n/a
	4	111120	14320	*23.598*	*11.319*	n/a	n/a	n/a	n/a
- 1.16	1	15522	21474	7451.9	19258	6745.4	22429	2623.2	7681
	2	104820	20895	61193	30836	6171.8	25592	4318.2	6123.2
	3	92176	11574	37613	8519.8	783.16	5136.4	3225.1	2405.6
	4	110470	13464	25890	10785	1091.9	5139.1	2915.8	1662.2
	5	75700	11576	17932	10397	942.26	6653.9	2838.2	1974.6
	6	92368	8161.6	19721	7790.6	629.25	4714.2	3069.4	1787.4
- 3.58	1	n/a	n/a	260080	360390	4.90E+06	2.77E+06	7514.8	8105.7
	2	n/a	n/a	101740	117150	1.28E+06	628390	3008.4	926.91
	3	n/a	n/a	98434	69260	5911.8	19904	2942.5	2479.5
	4	n/a	n/a	36565	14223	2103.6	9368.9	2355.3	1767
	5	n/a	n/a	19221	6772.1	989.83	6086.6	2096.7	1666.6
	6	n/a	n/a	7028	7207.7	-296.12	3159	1598.9	1610.9
- 6.36	1	93308	40565	113090	95076	94514	150000	17711	30643
	2	104920	26338	63350	26589	11360	16602	2098.6	6687.3
	3	6106.8	15100	-862.95	12225	4750.6	18116	-2912.3	5304.7
	4	95549	9788.1	32485	15468	5807.2	14140	-243.65	3432.6
	5	76434	16810	25031	16975	4499.8	11912	-676.16	3504.8
	6	118630	21679	22169	14942	2606.1	7671.4	-383.66	3396.8

Supplementary Movie Legends:

Supplementary Movie S1: Manipulation of multi-criteria voxelgrams to delineate the hippocampal formation (HIF)

Supplementary Movie S2: Three-dimensional volume reconstruction of high Zn and Fe concentrations in the mouse midbrain. Data reconstructed using ParaView.

Supplementary Movie S3: Three-dimensional volume reconstructions of tyrosine hydroxylase distribution (as ^{173}Yb and a proxy for dopamine) and Fe identifies areas where high Fe and dopamine colocalize.
